# Atypical location of primary cardiac lymphoma in the left heart with atypical clinical presentation: A case report and literature review

**DOI:** 10.3389/fsurg.2022.1036519

**Published:** 2023-01-16

**Authors:** Yongjia Qiang, Kuan Zeng, Bin Zhang, Ruicong Guan, Yuqiang Liu, Zhuxuan Liu, Haohua Xu, Xinyi Zhang, Yanting Ren, Baoping Deng, Yanqi Yang

**Affiliations:** ^1^ Department of Cardiovascular Surgery, Sun Yat-sen Memorial Hospital, Sun Yat-sen University, Guangdong, China; ^2^ Guangdong Provincial Key Laboratory of Epigenetics and Gene Regulation of Malignant Tumors, Sun Yat-sen Memorial Hospital, Sun Yat-sen University, Guangdong, China; ^3^ Department of Pathology, Sun Yat-sen Memorial Hospital, Sun Yat-sen University, Guangdong, China; ^4^ Department of Vascular Surgery, The Fifth Affiliated Hospital, Southern Medical University, Guangdong, China; ^5^ Department of Cardiothoracic Surgery, University Hospital, Linköping University, Linköping, Sweden

**Keywords:** primary cardiac tumors, lymphoma, biopsy, chemotherapy, immunotherapy, complete remission, case report

## Abstract

**Background:**

Primary cardiac lymphoma (PCL) is a rare and aggressive cardiac tumor with very poor prognosis that occurs mostly in the right cardiac cavity. Early diagnosis and treatment may improve its prognosis. In the present report, we describe the diagnosis and treatment of a primary cardiac diffuse large B-cell lymphoma (PC-DLBCL) with atypical location and clinical presentation. Additionally, a literature review was conducted to summarize the current knowledge of the disease.

**Case Presentation:**

A 71-year-old man visited his local hospital because of syncope, recurrent chest tightness, shortness of breath, palpitations, and profuse sweating for more than 20 days. Chest radiography revealed a mediastinal mass. Cardiac computed tomography (CT) showed multiple enlarged mediastinal lymph nodes. Transthoracic echocardiography (TTE) showed a cardiac mass in the posterior–inferior wall of the left atrium. He was then transferred to our hospital for positron emission tomography-CT (PET-CT) which showed active uptake of fluorodeoxyglucose both in the cardiac mass and in the multiple enlarged mediastinal lymph nodes. Biopsy of the enlarged mediastinal lymph nodes was carried out by using video-assisted thoracic surgery (VATS) technique, and pathological examination confirmed the subtype of PC-DLBCL, Stage IV, NCCN IPI 3. Therefore, the patient received a combination of chemotherapy and immunotherapy with R-CDOP (rituximab, cyclophosphamide, liposome doxorubicin, vincristine, and prednisone). After four courses of treatment in 4 months, the cardiac lymphoma and the enlarged mediastinal lymph nodes achieved complete remission with mild side effects of the chemotherapy.

**Conclusion:**

Early diagnosis and a precise choice of chemotherapy and immunotherapy based on cardiac imaging and pathological examination may improve the prognosis of PC-DLBCL in an atypical location.

## Introduction

Cardiac lymphoma is a rare condition and may be classified as primary and secondary types. The most frequently seen type is secondary cardiac lymphoma. Primary cardiac lymphoma (PCL) accounts for only about 1.3% of all primary cardiac tumors and 0.5% of extra nodal lymphomas (1, 2). PCL only invades the heart or pericardium (3). Diffuse large B-cell lymphoma (DLBCL), a subtype of non-Hodgkin's lymphoma, is considered the most common type of PCL, and it mostly occurs in the right atrium (4). The main clinical manifestation of patients with PCL is cardiac symptoms caused by myocardial infiltration of lymphoma, such as arrhythmias, heart failure, and chest pain (3, 4). There is no specific biomarker yet for the early diagnosis of PCL. Pathological examination on tumor biopsy or metastatic tissue may provide definite diagnosis. However, the biopsy procedure itself is associated with potential risks such as major bleeding, atrial perforation, chordal rupture, and arterial or pulmonary embolism, which makes early diagnosis of the tumor challenging.

Currently, chemotherapy combined with immunotherapy is recommended as the first-line treatment for lymphoma in the early stage instead of surgical treatment (5). Early diagnosis and a timely administered treatment are paramount to improve the survival rate (1).

In the present report, we describe the diagnosis and treatment of a patient with PCL in the left atrium and discuss the relevant literature to summarize current knowledge of the disease.

## Case presentation

A 71-year-old man who was a heavy smoker visited the emergency clinic at his local hospital because of syncope, recurrent chest tightness, dyspnea, palpitations, and sweating for more than 20 days. Electrocardiography (ECG) showed sinus rhythm with a first-degree atrioventricular (AV) block. Chest radiography revealed a mediastinal mass. The results of brain magnetic resonance imaging (MRI) and coronary angiography (CAG) were unremarkable. Cardiac computed tomography (CT) demonstrated multiple enlarged mediastinal lymph nodes and a low-enhanced solid lesion in the posterior–inferior wall of the left atrium, which was subsequently confirmed by transthoracic echocardiography (TTE).

**Figure 1:**
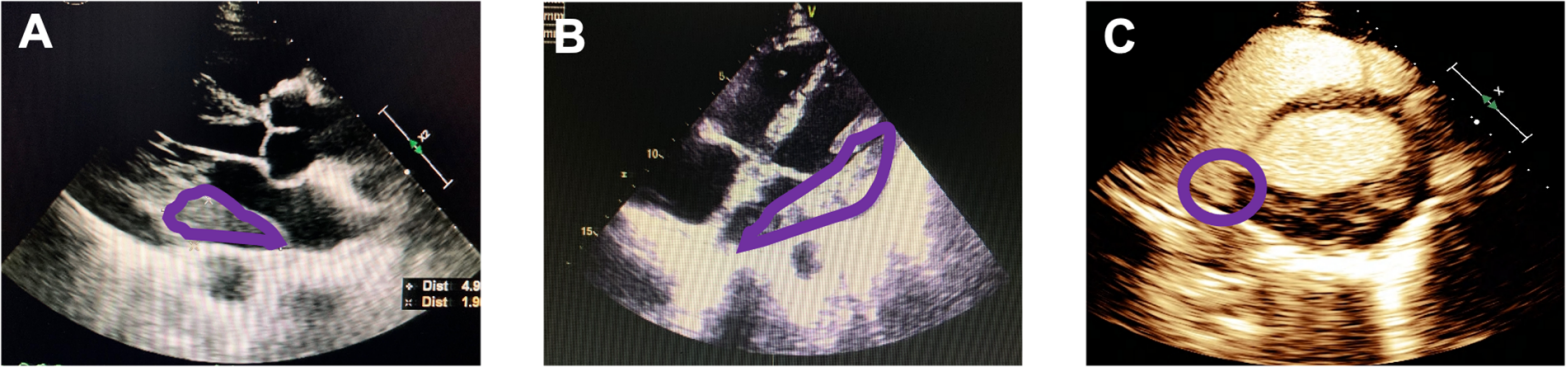
Echocardiography imaging: (**A**) parasternal long axis section; (**B**) four-chamber section. An intracardiac echogenic mass (in the purple circles) 50 mm × 67 mm × 19 mm was visualized at the left atrioventricular junction outside the cardiac cavity. (**C**) Left ventriculography and myocardial contrast echocardiography showed an abundance of contrast agent filling into left ventricular and the mass.

**Figure 2:**
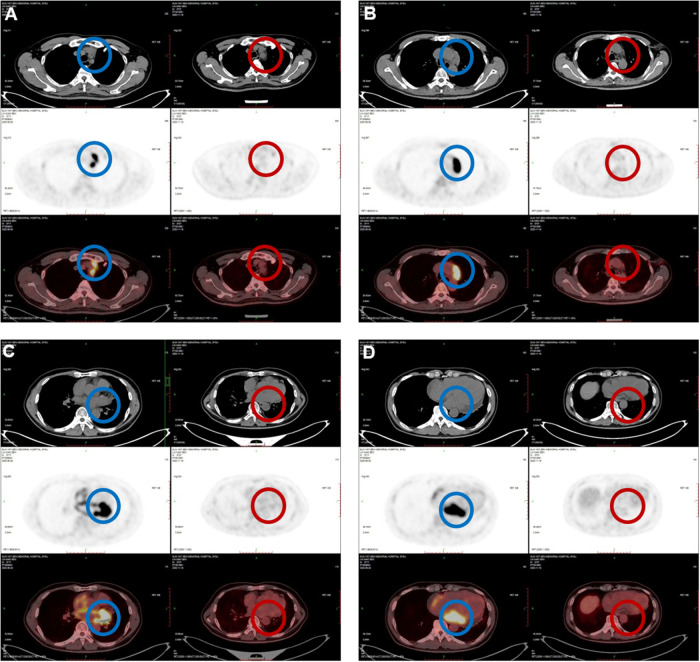
PET-CT imaging: (**A**) active FDG uptake of enlarged mediastinal multiple lymph nodes (SUVmax: 27.3) before treatment (in the blue circles); (**B**–**D**) active FDG uptake of the cardiac mass (SUVmax: 28.7) in the left atrium at the left AV junction (in the blue circles); (**A**–**D**) after treatment, the previous cardiac mass and enlarged mediastinal lymph nodes had disappeared completely (in the red circles).

**Figure 3:**
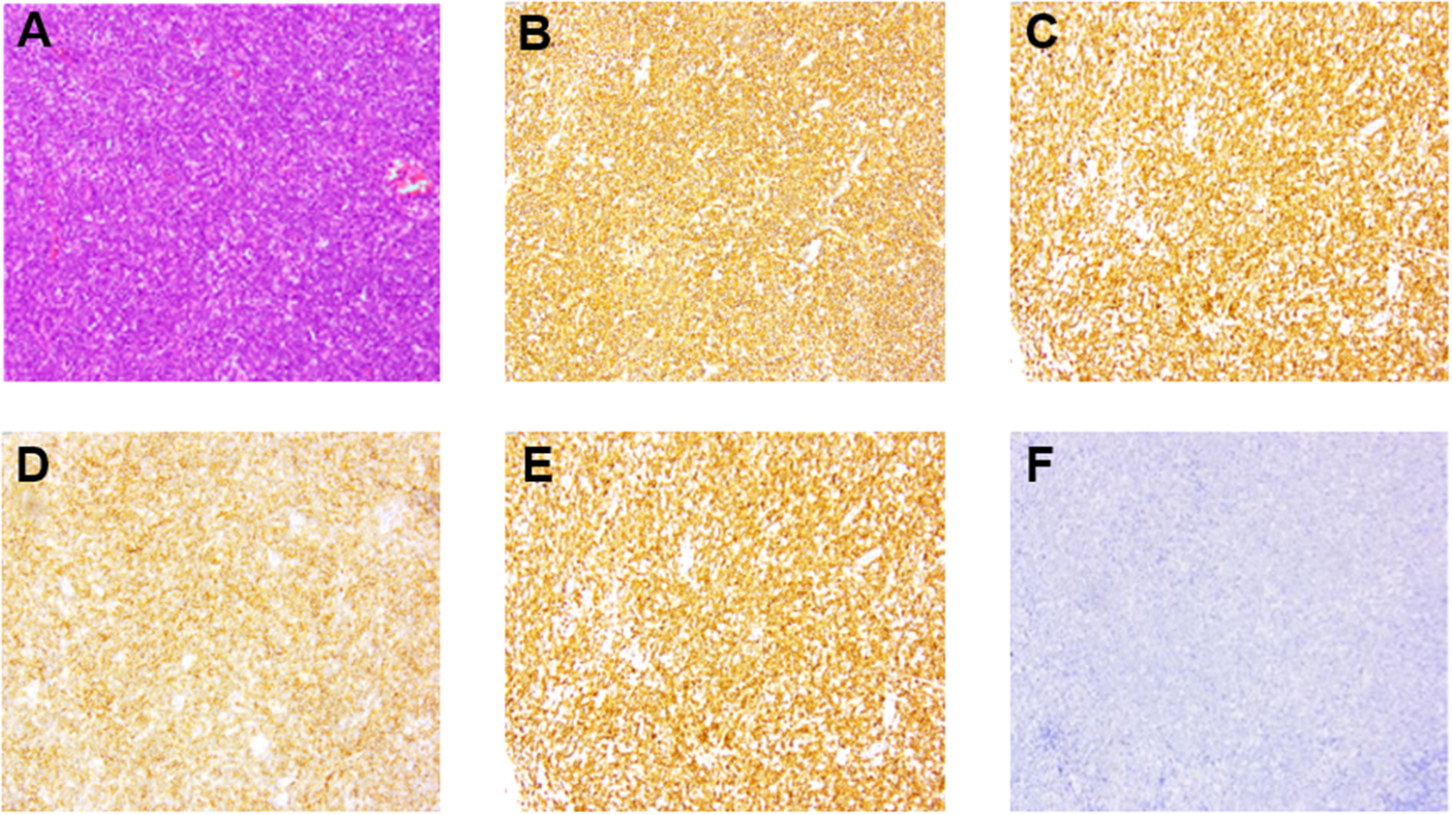
Pathological changes of specimen from the enlarged mediastinal lymph nodes. Medium-large heterotypic lymphoid cells showed diffuse and flaky hyperplasia. (**A**) (Hematoxylin and eosin stain, ⊆100) the cells were round/oval; the cytoplasm was red stained; the nuclear chromatin was rough, and the mitotic figures were abundant. (**B**–**E**) (Immunohistochemical stain, ⊆100) CD20 and CD79a high expression suggested B-cell origin. CD10 and BCL6 positive expression suggested the germinal center B-cell (GCB) type. (**F**) Fluorescence *in situ* hybridization (FISH) suggested EBER negative expression.

The patient was then transferred to our hospital for further evaluation and treatment. Routine physical examination and laboratory examination on admission were unremarkable. Holter ECG record presented sinus rhythm with first-degree AV block and paroxysmal atrial fibrillation. Cardiac CT scanning showed a small amount of pericardial effusion and pleural effusion on the left side. The solid echo mass on TTE was 50 mm × 67 mm × 19 mm in size and located in the left atrium at the AV junction without an obvious boundary to the adjacent cardiac tissues. The echo density of the mass was uneven. Left ventriculography and myocardial contrast echocardiography showed an abundance of contrast agent filling into left ventricular and the mass (Figure 1). PET-CT showed that the above mass was located in the posterolateral wall of the left atrium and left ventricle at the AV junction, measuring 61 mm × 26 mm with active uptake of fluorodeoxyglucose (FDG) (SUVmax: 28.7), which was indicative of lymphoma. Multiple enlarged mediastinal lymph nodes with active uptake of FDG (SUVmax: 27.3) were seen in neck IV and station 2L, 3a, 4, and 5 of the mediastinum, indicating metastasis (Figure 2). A small lymph node with slightly active uptake of FDG (SUVmax: 2.8) was seen in the right hilum which was considered as reactive changes. The above imaging findings suggested that the mass was a PCL. Mediastinal lymph node biopsy was carried out with video-assisted thoracic surgery (VATS) through a 3-cm–long incision in the fifth intercostal space on the mid axillary line. A piece of tissue measuring 3.5 cm × 3.0 cm × 0.6 cm was removed using an electro-diathermia knife. Pathological examination of the specimen was completed by using conventional H&E staining and immuno-histochemistry staining. The histologic features showed that the normal structure of the lymph node had been destroyed and medium-to-large heterotypic lymphoid cells showed diffuse hyperplasia. These heterotypic cells were round/oval with red-stained cytoplasm and active mitosis (Figure 3A). Immunohistochemistry showed positive results for CD20 (+++), CD79a (+++), CD19 (+++), mum-1 90% (+), CD10 80% (+), bcl-2 95% (+), bcl-6 95% (+), c-myc 25% (+), p53 40% (+), and Ki67 80% (+), and negative results for CD3 (−), CD5 (−), CD21 (−), CD23 (−), CD30 (−), ALK (−), CyclinD1 (−), and CD38 (−), and PD-L1 (22C3) expression of 35% the tumor cells. (Figure 3B–E). In situ hybridization remained negative for Epstein–Barr virus (EBV)-encoded small RNAs (EBERs). The FISH results showed BCL6 (+), MYC (−), and BCL2/IGH (−), which ruled out double- and triple-hit high-grade B-cell lymphoma (Figure 3F). The bone marrow aspirate and biopsy showed active bone marrow hyperplasia with normal trilineage hematopoiesis. A diagnosis of primary cardiac diffuse large B-cell lymphoma (GCB subtype, stage IV, NCCN-IPI 3) was established on the basis of the pathological evidence.

The patient was given a combined chemo-immunological therapy with R-CDOP (rituximab, cyclophosphamide, liposome doxorubicin, vincristine, and prednisone), which was decided upon after a multidisciplinary consultation by hematologists, oncologists, cardiologists, and cardiac surgeons. The patient received a single treatment course each month for 4 months. He was closely monitored during the chemo-immunotherapy, and an emergency plan was in place to deal with possible complications such as arrhythmia and cardiac rupture.

**Figure 4:**
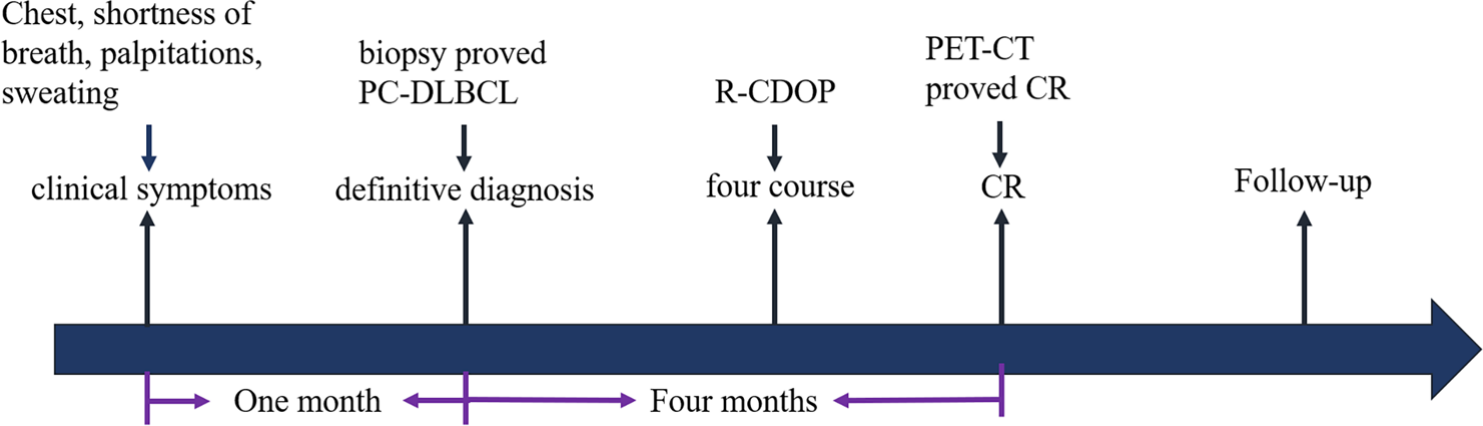
Flow chart of the patient's clinical course: the patient’s timeline of the onset of clinical symptoms, definitive diagnosis, treatment, and tumor response. CR, complete response.

After 4 months of the treatment, the PCL and the enlarged mediastinal lymph nodes achieved complete remission as seen on the follow-up PET-CT after the treatment (Figure 2). No obvious side effect of the therapy was observed during the course of treatment. The clinical course of the patient is presented in Figure 4. Further follow-up is required to ascertain the long-term outcome.

## Discussion and literature review

### Epidemiology

PCL is rare malignant cardiac tumor associated with low incidence and poorer prognosis than other primary malignant cardiac tumors such as sarcomas and myxoma (6). It accounts for about 1.3% of primary cardiac tumors and 0.5% of extra nodal lymphomas (1, 2). Most PCL are derived from B cells, and a few are derived from T cells (7, 8). Furthermore, PC-DLBCL is considered the most common histologic subtype of PCL, accounting for approximately 63%–85% of all PCLs (9–12).

There are no lymphoid nodes in the heart, and the source of PCL is thought to come from the drainage of the epicardial lymph nodes (13). The pathogenesis of PCL is still unknown, but is possibly related to recurrent infection and immune dysfunction, including human immunodeficiency virus (HIV) infection, EB virus infection, congenital immunodeficiency, and allogeneic bone marrow and solid organ transplantation (14).

### Clinical manifestation

The average age of patients with PCL at diagnosis has been reported to be 60 years. PC-DLBCL occurs more frequently in older male patients (11, 15–17). The most common symptoms are cardiac-related and dependent on its anatomic location in the heart, including arrhythmia resulting from compressed cardiac conducting system and symptoms of heart failure due to blockage of intra-cardiac blood follow. Lymphoma-related symptoms such as fever, night sweats, progressive weight loss, and other general systemic symptoms are uncommon in patients with PCL (18–21). Owing to the lack of specific symptoms and detection indices in the early stage, most patients are diagnosed at the middle and advanced stages.

Cardiac lymphomas mostly occur in the right cardiac cavity, mainly in the right atrium. One third of the cases occur in the pericardium. Less than 10% of cases occur in the left cardiac cavity. Valvular involvement is rare, which may be because of the limited lymphatic network in valvular tissue (4, 11). The PCL of our patient was located in the left atrium and different from most reported cases, which caused atypical clinical symptoms as described earlier.

### Differential diagnosis

An image of a cardiac mass in the left atrium can be attributed to thrombus or tumors that can be easily differentiated by using cardiac CT with contrast or by PET-CT. Cardiac tumors can be classified as primary and secondary tumors according to their origin. The tumors that most frequently metastasize to the heart are bronchogenic tumors, lymphomas, breast cancer, and melanoma (22, 23). The early clinical manifestations of the secondary or metastatic cardiac tumors are mostly symptoms of the primary tumor before cardiac-specific symptoms are presented.

The most commonly seen primary cardiac tumors in the left atrium are myxoma, with typical imaging characteristics such as clear border, lobular or regularly oval in shape, and being totally intra-cardiac with a pedicle attached to the atrial wall (24, 25). Their typical appearance on echocardiography makes it easy to differentiate from PCL (9).

Sarcomas are the most common primary cardiac malignancies, accounting for about 95%, of which angiosarcomas comprise 37% of all cases (17, 26). Angiosarcomas mostly originate in the right atrium and are predominantly located in the intramural space, which is associated with rapid growth, strong invasiveness, and broad-based cauliflower-like or nodular with surface bleeding and exudation. The characteristic imaging findings of sarcoma are invasive pericardial mass, pericardial thickening, and hemorrhagic pericardial effusion. Cardiac CT showed a low-density mass and heterogeneously enhanced. The tumor appears as heterogeneous signal intensities due to necrosis or intertumoral hemorrhage on MRI, and it shows obvious enhancement and “sunray” appearance, which expresses as a linear enhancement along the vascular spaces (27, 28). In the present case, the PCL was located in the left atrium at the AV junction without obvious boundary to the adjacent cardiac tissues, and the echo density of the mass was uneven on TTE. Cardiac CT demonstrated multiple enlarged mediastinal lymph nodes and a low-enhanced solid lesion in the left heart without the typical “sunray” signs of angiosarcoma.

### Diagnostic approach

According to the World Health Organization's 2015 diagnostic criteria, PCL can be diagnosed if it meets one of the following criteria: (i) primary lymphoma of the heart or pericardium; (ii) lymphoma with first cardiac-related symptoms; and (iii) lymphoma dominated by cardiac mass (29). However, its clinical manifestations are not significantly different from ordinary chest and heart diseases in the early stage, and little attention was paid to the disease. Therefore, PCL is obviously not the primary consideration for a patient who presents to the clinic with chest pain or dyspnea (4, 11).

Clinical imaging examination may often provide more information for differential diagnosis of a cardiac mass. In our case, the cardiac CT showed a cardiac mass with enlarged mediastinal lymph nodes, which showed active uptake of FDG with an SUVmax of 28.7 on PET-CT, thereby suggestive of PCL associated with metastasis of mediastinal lymph nodes.

When imaging examination indicates that there is a large pericardial effusion, pericardiocentesis and thoracoscopic pericardial window can be performed, which is helpful to early diagnosis and symptom relief by lymphoma cell detection from pericardial fluid. However, cytology is often nonspecific, which needs to be combined with the histological examination of pericardial biopsies to make the final diagnostic. When cytological examination is difficult to achieve, biopsy of the cardiac mass or metastatic lymph nodes is the quickest and most reliable method for pathological diagnosis of PCL such as thoracotomy (open biopsy), mediastinoscopy, TEE-guided biopsy, and endomyocardial transvenous biopsy. A questionable diagnosis is achieved in a patient when endomyocardial transvenous biopsy is performed, which has a positive rate of 50%. Open biopsy is not the optimal choice unless the tumor has already seriously threatened the life of the patient, even though it has a high positive rate of 100%. Mediastinoscopy and TEE-guided biopsy can also provide the most reliable results, which has a positive rate of 100%. However, the biopsy procedure itself is associated with potential risks such as major bleeding, atrial perforation, chordal rupture, and arterial or pulmonary embolism, which makes early diagnosis of the tumor challenging. Selection of optimize biopsy modality relies on the accurate localization of the mass by radiographic findings and the patient's tolerance of the therapy (20).

In addition, 18F-FDG PET-CT is also an important means to diagnose cardiac lymphoma, which can noninvasively detect the metabolic activity of tumors and stage lymphoma more accurately. As described by the Warburg effect, compared with normal cells, tumor cells tend to provide energy through glycolysis in an aerobic environment (30). The degree of aerobic glycolysis reflects tumor activity and malignancy, which is reflected by the SUV on PETCT. Cardiac lymphoma is highly metabolic with high F18-FDG uptake (31, 32), which is significantly higher than that of patients with other cardiac malignant tumors such as metastatic tumors and sarcomas and benign tumors (33, 34). The SUVmax of cardiac lymphoma is greater than 10. Currently, 18F-FDG PET/CT is an important tool for early diagnosis, guiding treatment, restaging after treatment, and evaluation of efficacy and prognosis of lymphoma (35, 36), and it is also well complementary to pathological findings.

### Treatment and outcomes

According to a previous study, an average survival time of all PCL is 215 days (260.1 days for T-cell lymphoma and 217.9 days for *B*-cell lymphoma) (4). The currently suggested strategies for PCL treatments are surgery, chemotherapy, radiotherapy, and immunotherapy (5, 37, 38). However, there is still no gold standard of care for PCL.

The CHOP (cyclophosphamide, doxorubicin, vincristine, prednisone) regimen remains the classical choice for the treatment of DLBCL. Rolla et al. reported 66 patients with PCL, 31 of whom received a CHOP-based chemotherapy regimen with a mean median survival of 7 months (39). With the availability and widespread use of rituximab (R), the combination of immunotherapy with chemotherapy (R-CHOP regimen) has become the first line of treatment for DLBCL instead of surgical treatment, regardless of the tumor stage (40–43). The long-term outcome of the R-CHOP regimen is superior to that of CHOP alone (44, 45). Chemotherapy combined with locoregional radiotherapy for early-stage non-cardiac DLBCL can lead to satisfactory treatment outcomes and favorable outcomes (46, 47). For patients with PCL, there is insufficient evidence that this approach can improve outcomes (48). Palliative surgery or total resection can rapidly relieve patient symptoms, clarify the type of pathology, and provide the basis for chemotherapy. However, there is no evidence that surgery or heart transplantation can improve patient outcomes (49, 50).

Here, we used liposome doxorubicin to replace doxorubicin, owing to its satisfactory therapeutic effect and prognosis. The existence of liposomes can significantly reduce adverse cardiac reactions of anthracycline drugs and penetrate the blood–brain barrier and blood–testis barrier (51–54). And the drug concentration in tumor tissue can reach as 20–60 times higher than in normal tissue, which can effectively improve the prognosis of moderate- and high-risk DLBCL patients, especially those with multiple extra nodal involvement and large mass like the present case (55, 56).

## Conclusion

The results of our study highlight the importance of routine health screening for cardiac disease in people, even in the absence of symptoms. Early diagnosis and a precise choice of chemotherapy and immunotherapy based on cardiac imaging and pathological examination may improve the prognosis of PC-DLBCL in an atypical location. And further research is required to evaluate the long-term outcome of the treatment.

## Data Availability

The original contributions presented in the study are included in the article/Supplementary Material, further inquiries can be directed to the corresponding author/s.
